# Supplementary material for: Autoantibodies stabilize neutrophil extracellular traps in COVID-19
Source: JCI Insight. 2021 Aug 9;6(15):e150111. doi: 10.1172/jci.insight.150111 (PMC8410057; doi:10.1172/jci.insight.150111)
Supplement: Supplemental data [file jciinsight-6-150111-s204.pdf]

Supplementary Materials

Autoantibodies stabilize neutrophil extracellular traps in COVID-19

Zuo *et al.*

Supplementary Table 1: Correlation of anti-NET IgG/IgM with antiphospholipid antibodies in COVID patients

	Anti-NET IgG			Anti-NET IgM	
	r	p		r	p
**Antiphospholipid antibodies**					
aCL IgG	0.45	****	aCL IgM	0.34	****
aβ_2GPI IgG	0.0004	ns	aβ_2GPI IgM	0.18	*
aPS/PT IgG	0.27	***	aPS/PT IgM	0.44	****

aCL, anticardiolipin antibodies; aβ_2GPI, anti-beta-2 glycoprotein I antibodies; aPS/PT, anti-phosphatidylserine/prothrombin antibodies
ns=not significant; *$p<0.05$, ***$p<0.001$, and ****$p<0.0001$

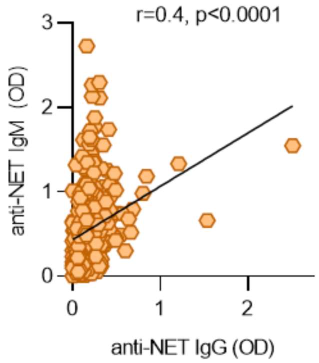

Supplementary Figure 1: **Association of anti-NET IgG with anti-NET IgM.** Anti-NET IgG and anti-NET IgM were compared for 328 COVID patients. Spearman's correlation coefficient was calculated and is shown in the panel.

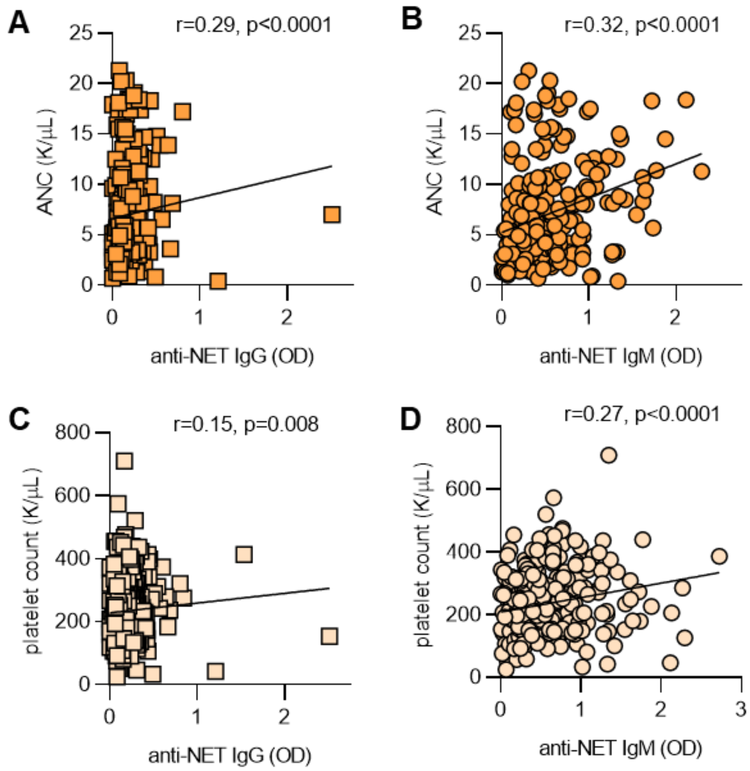

Supplementary Figure 2: **Association of anti-NET IgG/IgM with absolute neutrophil count (ANC) and platelet count. A-B,** Anti-NET IgG and IgM levels were compared to ANC on the same day as the research sample (n=266 COVID-19 patients). **C-D,** Anti-NET IgG and IgM levels were compared to platelet count on the same day as the research sample (n=315 COVID-19 patients). Spearman's correlation coefficients were calculated and are shown in the panels. ANC=absolute neutrophil counts.

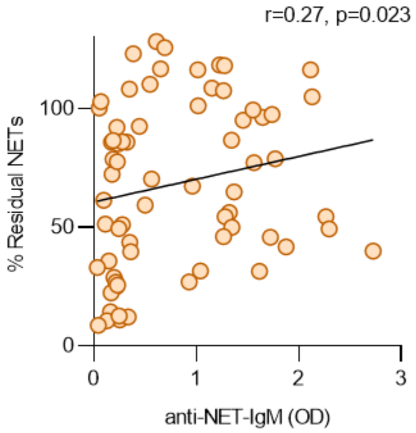

Supplementary Figure 3: **Anti-NET IgM correlates with impaired NET degradation.** Freshly-induced NETs were incubated with COVID sera, and percent residual NETs was determined for each sample after 90 minutes. Percent residual NETs was normalized against the mean of untreated NETs. Correlation with anti-NET IgM was determined by Spearman's method.
